# In vitro antimicrobial activity of plants in Acute Otitis Externa

**DOI:** 10.1016/S1808-8694(15)30761-8

**Published:** 2015-10-19

**Authors:** Janaina Cândida Rodrigues Nogueira, Margareth de Fátima Melo Diniz, Edeltrudes O. Lima

**Affiliations:** 1 MSc, MD; 2 PhD. Head of the centro de ciências de saúde /UFPB; 3 PhD. Professor of Pharmachology - UFPB

**Keywords:** microbiology, otitis external, plants

## Abstract

Acute Otitis Externa is an inflammation of the outer auditory meatus, and according to popular saying, medicinal plant extracts can be used in its treatment.

**Aim:**

to assess the in vitro antimicrobial activity of the following plants: *Aleolanthus suaveolens; Caryophyllus aromaticus; Cymbopogon citratus; Matricaria chamomila; Pithecellobium avaremotemo; Plectranthus amboinicus* and *Ruta graveolens* on the germs that cause otitis externa.

**Materials and Methods:**

the minimum inhibitory concentration of extracts and oils from these plants was obtained from otitis externa samples.

**Results:**

*Staphylococcus aureus* in 10 cultures, *Pseudomonas aeruginosa* in 8, *Pseudomonas aeruginosa* and *Staphylococcus aureus* together in 5 cultures and *Candida albicans* and *Candida krusei* in 4 cultures. *P. aeruginosa* was resistant to all oils and extracts tested; extracts from *A. suaveolens, P. avaremotemo* and *R. graveolens* were inactive; the essential oil from *C. aromaticus* and M. chamomila were active against 3 strains of *S. aureus* and the Candida strains; seven of the *S. aureus* strains were sensitive to the *P. amboinicus* extract; however, the oil was inactive against 4 *S. aureus* strains and the Candida strains were sensitive to the *R. graveolens* essential oil.

**Conclusion:**

depending on the etiological agent, some plants presented satisfactory results, however we still need more detailed studies in order to better use these plants.

## INTRODUCTION

Otitis externa is basically an inflammation on the external acoustic meatus skin, frequently associated with a secondary bacterial infection and/or fungal infection on the macerated skin of the subcutaneous tissue.^1^^,^^2^ It can be classified into diffuse, which is a dermatitis, when there is a reduction in wax production, epithelial scaling and edema; or localized, in this case it is a furuncle, caused by an infection in the pilosebaceous foliculum.^1^^,^^2^ In folk's medicine, it is common to use medicinal Brazilian plants such as arruda, macassá, barbatimão, among others, depending on the region, without however, any scientific study justifying its use. The World Health Organization advocates the use of medicinal plants, especially in public health care programs in developing countries.^3^^,^^4^ The plants can be used as infusion, macerated, as juice, tinctures, infiltrates or patches, for the treatment of diseases.^3^^,^^5^ They have many secondary metabolic pathways which will create compounds that include alkaloids, flavonoids, isoflavonoids, tannins, coumarins, glycosides, terpenes, polyacetylenes, which are specific to certain families, genera or species and that have many functions.^5^ Otitis externa is preferably treated topically, considering antimicrobial resistance stemming today as a risk to its therapeutic efficacy. Considering the prevalence and morbidity of this disease and the very possibility of having other means of treatment, this study aimed at assessing the in vitro antimicrobial activity of these plants' extracts and essential oils on microorganisms taken from patients with acute otitis externa.

## MATERIALS AND METHODS

Twenty-seven patients with clinical diagnosis of AOE (Acute Otitis Externa) without ear drum perforation nor prior use of medication,^6^ were selected, regardless of gender and age, during a period of three months, and we collected material from the involved ear, by using a Swab, following the standards and guidelines established for research projects involving human beings - Resolution # 196/1996 - Brazilian Ministry of Health.^7^ The study was approved by the ethics committee of the Lauro Wanderlei University Hospital, protocol # 341, and the patients signed an informed consent form in order to take part in the project. In order to check for antibacterial activity on the plants' extracts and essential oils, we isolated and identified the bacterial species, according to microbiology routines,^8^, ^9^, ^10^ and the samples were kept in agar Miller Hinton (Difco Laboratories Ltd.). From otomycosis we isolated and identified yeasts by macro and micro morphological and biochemical characteristics.^11^, ^12^, ^13^, ^14^, ^15^ The strains were kept in 2% agar Saboraud dextrose (ASD) (Difco Laboratories Ltda), stored at room temperature and at 4°C. The extracts and essential oils used in the antimicrobial activity assays were obtained from the four plant species included on Chart 1. In order to assess antimicrobial activity, we tested the extracts in the following concentrations: 5000, 2500, 1250, 625, 313, 156 and 78μg/ml, solubilized in dimethyl-sulphoxyde-DSO, at a maximum rate of 10% and the essential oils were tested in the concentrations of 8, 4, 2, 1, 0.5 and 0.25%, according to Allegrini et al's technique. 1973.^16^^,^^17^

**Chart 1:** Plant species and respective products used to assess their antimicrobial activity on bacteria and fungi.

Gender/ Species Family	Common name	Obtainment	Plant part	Product tested
*Aleolanthus suaveolens Spreng. Labiatae*	Macassá	Obtained from a natural products' store	Leaf	Extract
*Caryophyllus aromaticus L. Myrtaceae*	Cravo-da-índia	Obtained from a natural products' store	Leaf	Base oil
*Cymbopogon citratus Stapf. Gramineae*	Capim-santo	Medicinal plants garden - UFPB	Leaves	Base oil
*Matricaria chamomila (L) Rausch Asteraceae*	Camomila	Obtained from a natural products' store	Leaves and flowers	Base oil
*Pithecellobium avaremotemo Mart. Fabaceae (Leguminosae)*	Barbatimão	Obtained from a natural products' store	Stem's skin	Extract
*Plectranthus amboinicus Lour Lamiaceae*	Hortelã da folha grossa	Medicinal plants garden - UFPB	Leaves	Base oil/extract
*Ruta graveolens L. Rutaceae*	Arruda	Medicinal plants garden - UFPB	Leaves	Base oil/extract

We carried out antibacterial and antifungal activities assays to determine the minimum inhibitory concentration MIC of extracts and essential oils by the diffusion in solid medium method - cavity-plate process.^9^^,^^16^ Control was carried out by antibiogram.

## RESULTS

We observed *Staphylococcus* aureus in 10 cultures (37%), *Pseudomonas aeruginosa* in 8 cultures (29.6%), *Pseudomonas aeruginosa and Staphylococcus* aureus together in 5 cultures (18.5%), and Candida in 4 cultures, (14.9%), in all cases, they were associated with Gram Positive and Gram Negative Bacteria.

In the present investigation we assessed the in vitro antibacterial and antifungal activity of extracts and/or essential oils of the following medicinal plants: macassá, barbatimão, cravo-da-índia, capim-santo, camomila, hortelã da folha grossa and arruda against twelve strains of *P. aeruginosa*, eight strains of *S. aureus*, one strain of *C. albicans* and one strain of *C. krusei*.

One can see that all fungal and bacterial species investigated resisted the extract of *A. suaveolens, Pithecellobium avaremotemo* and *Ruta graveolens*.

On Table 1 we list the results of antibacterial activity for the essential oil from *C. aromaticus*. Pseudomonas genera was completely resistant to this essential oil in all concentrations. In the same concentration of 4% it was active against the growth of three strains of Staphylococcus and two strains of Candida. We can see that *C. albicans* and *C. Krusei* were sensitive in concentrations of up to 1%, with 10 mm in diameter inhibition halos.

**Table 1:** Average value of inhibition halos (mm) of MIC assessment of *C. aromaticus* essential oil against bacteria and fungus in solid medium.

Microorganisms	Essential oil from *C. aromaticus* (%)						Control Microorganisms
	8	4	2	1	0,5	0,25	
*S. aureus (1)*	13	10	8	0	0	0	+
*S. aureus (2)*	0	0	0	0	0	0	+
*S. aureus (3)*	12	3	0	0	0	0	+
*S. aureus (4)*	11	0	0	0	0	0	+
*S. aureus (5)*	0	0	0	0	0	0	+
*S. aureus (6)*	10	8	0	0	0	0	+
*S. aureus (7)*	13	10	0	0	0	0	+
*S. aureus (8)*	12	10	0	0	0	0	+
*C. albicans (1)*	20	16	14	10	0	0	+
*C. krusei (2)*	22	18	15	10	0	0	+

+: Microorganism control in the culture medium without antimicrobial agent.

The essential oil from *M. chamomila* did not produce inhibition on Pseudomonas; however, in the concentration of 4%, it had an inhibitory activity on the growth of three strains of Staphylococcus and two Candida strains with inhibition halos varying between 10 and 12mm in diameter (Table 2).

**Table 2:** Mean values of MIC assessment inhibition halos (mm) from the essential oil of *M. chamomila* against bacteria and fungi, in solid medium.

Microorganisms	Essential oil from *M. camomila* (%)						Control Microorganisms
	8	4	2	1	0,5	0,25	
*S. aureus (1)*	0	0	0	0	0	0	+
*S. aureus (2)*	14	10	0	0	0	0	+
*S. aureus (3)*	0	0	0	0	0	0	+
*S. aureus (4)*	10	8	0	0	0	0	+
*S. aureus (5)*	0	0	0	0	0	0	+
*S. aureus (6)*	0	0	0	0	0	0	+
*S. aureus (7)*	15	10	0	0	0	0	+
*S. aureus (8)*	14	10	0	0	0	0	+
*C. albicans (1)*	14	10	0	0	0	0	+
*C. krusei (2)*	14	12	10	0	0	0	+

+: Microorganism control in a culture medium without antimicrobial agents.

*P. amboinicus* extract in the present study proved to be active against seven of the eight copies of Staphylococcus, with inhibition halos average of 13mm in diameter; however, it was not active against *P. aeruginosa* and *Candida ssp*. (Table 3). The oil from this plant was not very effective in this study, showing action against two strains of *S. aureus* and *C. krusei* strain in the concentrations of 8 and 4% for the bacterium and 8, 4 and 2% for the fungus, these data are shown on Table 4.

**Table 3:** Inhibition halos (mm) mean value from the MIC assessment of *P. amboinicus* extract against bacteria and fungi in solid medium

Microorganism	*P. amboinicus* extract (μg/ml)							Control Microorganism
	5000	2500	1250	625	313	156	78	
*S. aureus (1)*	0	0	0	0	0	0	0	+
*S. aureus (2)*	17	13	0	0	0	0	0	+
*S. aureus (3)*	20	15	12	0	0	0	0	+
*S. aureus (4)*	17	13	7	0	0	0	0	+
*S. aureus (5)*	15	12	8	0	0	0	0	+
*S. aureus (6)*	15	12	0	0	0	0	0	+
*S. aureus (7)*	18	14	12	0	0	0	0	+
*S. aureus (8)*	18	14	12	0	0	0	0	+
*C. albicans (1)*	0	0	0	0	0	0	0	+
*C. krusei (2)*	0	0	0	0	0	0	0	+

+: Microorganism control in a culture medium without bacteria.

**Table 4:** Mean value of inhibition halos (mm) from the MIC assessment of the *P. amboinicus* essential oil against fungi and bacteria in solid medium.

Microorganism	*P. amboinicus* essential oil (%)						Control Microorganism
	8	4	2	1	0.5	0.25	
*S. aureus (1)*	0	0	0	0	0	0	+
*S. aureus (2)*	0	0	0	0	0	0	+
*S. aureus (3)*	12	10	7	0	0	0	+
*S. aureus (4)*	10	0	0	0	0	0	+
*S. aureus (5)*	0	0	0	0	0	0	+
*S. aureus (6)*	12	10	0	0	0	0	+
*S. aureus (7)*	0	0	0	0	0	0	+
*S. aureus (8)*	0	0	0	0	0	0	+
*C. albicans (1)*	0	0	0	0	0	0	+
*C. krusei (2)*	14	12	10	0	0	0	+

+: Microorganism control in a culture medium without bacteria

The essential oil from *Ruta graveolens* extracts was assessed as to its antimicrobial action, because this plant has its popular use recognized in external ear pathology. The essential oil from *R. graveolens* at 4%, inhibited four Staphylococcus strains and all the Candida strains, with inhibition halos between 10 and 13 mm in diameter. These findings can be seen on Table 5; however, its extract did not show any activity.

**Table 5:** Inhibition halos mean value (mm) in MIC assessment of *R. graveolens* essential oil against bacteria and fungi, in solid medium.

Microorganism	*R. graveolens* essential oil (%)						Control Microorganism
	8	4	2	1	0,5	0,25	
S. aureus (1)	15	10	0	0	0	0	+
S. aureus (2)	10	0	0	0	0	0	+
S. aureus (3)	12	10	0	0	0	0	+
S. aureus (4)	15	10	0	0	0	0	+
S. aureus (5)	10	0	0	0	0	0	+
S. aureus (6)	16	12	10	8	0	0	+
S. aureus (7)	20	7	14	10	0	0	+
S. aureus (8)	10	8	0	0	0	0	+
C. albicans (1)	15	10	0	0	0	0	+
C. krusei (2)	17	13	10	0	0	0	+

+: Microorganism control in the culture medium without antimicrobial drug.

## DISCUSSION

Otitis externa is an infectious inflammatory process, which affects the external auditory meatus skin and usually has polymicrobial etiology.^2^ Hwang, Chu and Liu (2002)^18^ carried out a bacteriologic study in 161 patients and observed that *S. aureus* was as frequently found as *P. aeruginosa* in patients with otitis externa. These data ratify the findings of the present investigation, since there was no statistically significant difference between these bacteria. Kuczkowski et al. (2000)^19^, studied 55 cultures of patients with otitis externa and found a greater frequency of *Staphylococcus aureus, Pseudomonas aeruginosa* and *Proteus mirabilis*, similar to our findings; thus, we can consider that the main etiology associated with otitis externa is bacterial, and occasionally fungal.^18^ As far as fungi are concerned, the genus Candida was found in 4 cultures, associated with Gram-positive and Gram negative bacteria. Similar findings were reported by Ologe et al. (2002)^20^, when they investigated 141 patients with presumed otomycosis, they confirmed the disease in 76 (53.9%), and the fungi most frequently found were *Aspergillum sp* (63.4%), Candida (35.5%) and Mucor (1.3%).

Despite high technology and heavy investments from the pharmaceutical industry in the production of increasingly more potent antimicrobial drugs, microbial resistance is on the rise.^21^ The use of plant components with therapeutic ends has increased in Brazil. According to the World Health Organization, the use of plant components for therapeutic means has been on the rise in Brazil, and they also state that this can represent the best source of a variety of drugs.^21^^,^^22^ Nonetheless; these plants should not be used empirically, but rather based on controlled studies of efficacy and toxicity.

*A. suaveolens* - our macassá, although very much used in popular medicine for ear disorders, is not reported in the literature, both as antimicrobial drug as in terms of its analgesic and anti-inflammatory action that could justify its use, and in this study it was not efficient against the strains tested.

*P. avaremotemo* extract, although active against bacteria and fungi in many studies, did not prove to have any activity against external ear microorganisms.^23^ These findings may be associated with pathogen virulence and/or extract characteristics.

The activity of essential oils and plant extracts against bacteria and fungi is due to the presence of phyto components, which concentration may vary depending on a number of factors, such as plant growing, harvesting and also the means used to obtain the extract or the essential oil.^23^^,^^24^

It has been reported that essential oils are more effective against Gram positive bacteria than Gram negative, but there still is no scientific explanation for this fact.^21^ In the present study, the Gram negative P. aeruginosa bacteria resisted all tested oils, while the Gram positive S. aureus was sensitive to them, depending on the plant tested and its concentration.

The essential oil from *C. aromaticus* had an antimicrobial action against Gram positive bacteria, both in the literature studied as well as in the present investigation.^23^^,^^24^ We observed that the essential oil from *C. citratus* was not active against the bacteria and fungi used in this study; however, Araújo (2003)^25^ observed that this oil in the concentration of 8% was active against this fungi, but not against bacteria, as we found in this study.

The essential oil from *M. chamomila* inhibited the growth of *Staphylococcus aureus* and *Bacillus subtilis* and its hydro-alcoholic extract inhibited the growth of *Staphylococcus aureus, Streptococcus mutans*, Group *B Streptococcus, Streptococcus salivarius*, according to Jakovlev, 1983^26^. In numerous papers, A *M. chamomila* presented antimicrobial activity, especially against *P. aeruginosa*^27^ strains.

Catillo and Gonzälez (1999) observed that both the extract and the essential oil from *P. amboinicus*, harvested in Cuba, were active against Gram negative and Gram positive bacteria, as well as against yeasts and dermatophagoides, and the extract was more effective.^28^

According to Araújo (2003)25, the essential oil from *R. graveolens* was active against species of yeast, but not against *S. aureus*, similarly to the findings from Ross et al. (1980)^29^. However, Sá et al. (1995) noticed its activity against *P. aeruginosa, S. aureus, B. subtilis*^25^ strains. These differences can be credited to the essential oil concentration and the microbial strains tested.

Further and more comprehensive studies with medicinal plants in acute otitis externa are required, especially with those plants already advocated by popular medicine. However, it is worth stressing that this is the first approach carried out in the Northeast aiming at studying if any of these plants were effective against acute otitis externa causing agents, therefore we need further studies because this is a promising therapeutic alternative, especially for the needy population.

## CONCLUSION

*S. aureus, P. aeruginosa* and fungi of the genera Candida were the most frequently found microorganisms in the culture of patients with acute otitis external. *P. aeruginosa* was resistant to all tested extracts and essential oils. The essential oils from *C. aromaticus and M. chamomila* at 4% inhibited the growth of three *S. aureus* strains and of all *Candida strains*; such activity was seen at the concentration of 1%. The essential oil from *R. graveolens* at 4% inhibited *Candida strains* and four *S. aureus* strains; and the extract from *P. amboinicus* had inhibitory activity against seven *S. aureus* strains, and *Candida strains* were resistant. Some plants have a satisfactory effect depending on the etiological agent. We still need more comprehensive and detailed studies regarding the use of these plants, because they can represent a promising therapeutic alternative in the near future, especially in regards of the more needy population.
